# Drivers of sex differences in the South African adult tuberculosis incidence and mortality trends, 1990–2019

**DOI:** 10.1038/s41598-023-36432-6

**Published:** 2023-06-10

**Authors:** Mmamapudi Kubjane, Morna Cornell, Muhammad Osman, Andrew Boulle, Leigh F. Johnson

**Affiliations:** 1 Centre for Infectious Disease Epidemiology and Research, School of Public Health, University of Cape Town, Cape Town, South Africa; 2 Desmond Tutu TB Centre, Department of Paediatrics and Child Health, Faculty of Health Sciences, Stellenbosch University, Cape Town, South Africa; 3 School of Human Sciences, Faculty of Education, Health and Human Sciences, University of Greenwich, London, UK; 4 Western Cape Provincial Department of Health, Cape Town, South Africa

**Keywords:** Epidemiology, Statistics, Tuberculosis

## Abstract

Males have higher tuberculosis incidence and mortality rates than females. This study aimed to assess how sex differences in tuberculosis incidence and mortality could be explained by sex differences in HIV, antiretroviral treatment (ART) uptake, smoking, alcohol abuse, undernutrition, diabetes, social contact rates, health-seeking patterns, and treatment discontinuation. We developed an age-sex-stratified dynamic tuberculosis transmission model and calibrated it to South African data. We estimated male-to-female (M:F) tuberculosis incidence and mortality ratios, the effect of the abovementioned factors on the M:F ratios and PAFs for the tuberculosis risk factors. Over the period 1990–2019, the M:F ratios for tuberculosis incidence and mortality rates persisted above 1.0, and the figures reached 1.70 and 1.65, respectively, by the end of 2019. In 2019, HIV contributed greater increases in tuberculosis incidence among females than males (54.5% vs. 45.6%); however, females experienced more reductions due to ART than males (38.3% vs. 17.5%). PAFs for tuberculosis incidence due to alcohol abuse, smoking, and undernutrition, in men were 51.4%, 29.5%, and 16.1%, respectively, higher than females (30.1%, 15.4%, and 10.7%, respectively); the PAF due to diabetes was higher in females than males (22.9% vs. 17.5%). Lower health-seeking rates in males accounted for a 7% higher mortality rate in men. The higher burden of tuberculosis in men highlights the need to improve men’s access to routine screening and ensure earlier diagnosis. Sustained efforts in providing ART remain critical in reducing HIV-associated tuberculosis. Additional interventions to reduce alcohol abuse and tobacco smoking are also needed.

## Introduction

Globally, males experience higher tuberculosis incidence and mortality than females^1,2^. The male-to-female (M:F) tuberculosis incidence ratio varies by geographic region ranging between 1.1 and 2.5^1^. A meta-analysis of 39 prevalence surveys conducted in 28 countries estimated males to have 2.21 times higher tuberculosis prevalence than females^3^. Sex disparities in the burden of tuberculosis are driven by multiple factors, including socio-behavioural and biological differences that directly or indirectly affect the risk of exposure to *Mycobacterium tuberculosis*, acquiring latent infection, or developing active disease^4^. Biological hypotheses suggest that female sex hormones may protect against susceptibility to infection and the development of tuberculosis disease^5^. Males may be more exposed to additional risk factors for tuberculosis such as tobacco smoking and alcohol abuse^6–8^. Other conditions that increase susceptibility to tuberculosis disease include HIV, diabetes and undernutrition^9,10^. These risk factors increase the likelihood of developing tuberculosis by suppressing cell-mediated immunity^11,12^ and explain a considerable amount of the burden of tuberculosis at the population level^2^.

HIV, the most potent tuberculosis risk factor, is also distributed differently by sex, with a heavier burden among females than males^13^. However, compared to females, males are less likely to get tested for HIV and have lower antiretroviral therapy (ART) initiation rates^14^. Several studies have shown that the age-sex distribution of tuberculosis reflects that of the HIV epidemic^15–18^. However, limited studies have quantified the effect of the evolving HIV epidemic and the impact of the rollout of ART on the sex distribution of tuberculosis.

Several analyses have explored explanations for the excess burden of tuberculosis in men. Horton et al. showed that in Vietnam and Malawi, men had higher rates of tuberculosis incidence and longer delays to treatment^19^. Other studies have suggested that the frequent social contacts men have with other men^20^, combined with their higher rates of tuberculosis incidence^3^, likely amplifies their burden of tuberculosis^21,22^. The Global Burden of Disease Study (GBD) 2019 demonstrated the contribution of smoking, alcohol, and diabetes to sex disparities in tuberculosis mortality, showing that eliminating these risk factors would reduce the global tuberculosis mortality M:F ratios from 1.97 to 1.28^2^.

In South Africa, the male tuberculosis prevalence is approximately 1.6 times that in females^23^. However, limited analyses have evaluated how modifiable risk factors explain sex disparities at a population level. Understanding the factors that drive sex disparities and the overall burden of tuberculosis is essential for identifying where tuberculosis control efforts need to focus. The specific objectives of this study were to 1) quantify the effect of the evolving HIV epidemic and the impact of the rollout of ART on the sex distribution of tuberculosis incidence and mortality over the period 1990–2019; 2) estimate the sex-specific population attributable fraction (PAF) for undernutrition, smoking, alcohol, diabetes and HIV (2019); 3) to estimate the impact of sex differentials in a) tuberculosis health seeking b) HIV testing and ART initiation, c) social mixing patterns and d) tuberculosis treatment retention.

## Methods

### The tuberculosis model structure

**Figure 1:**
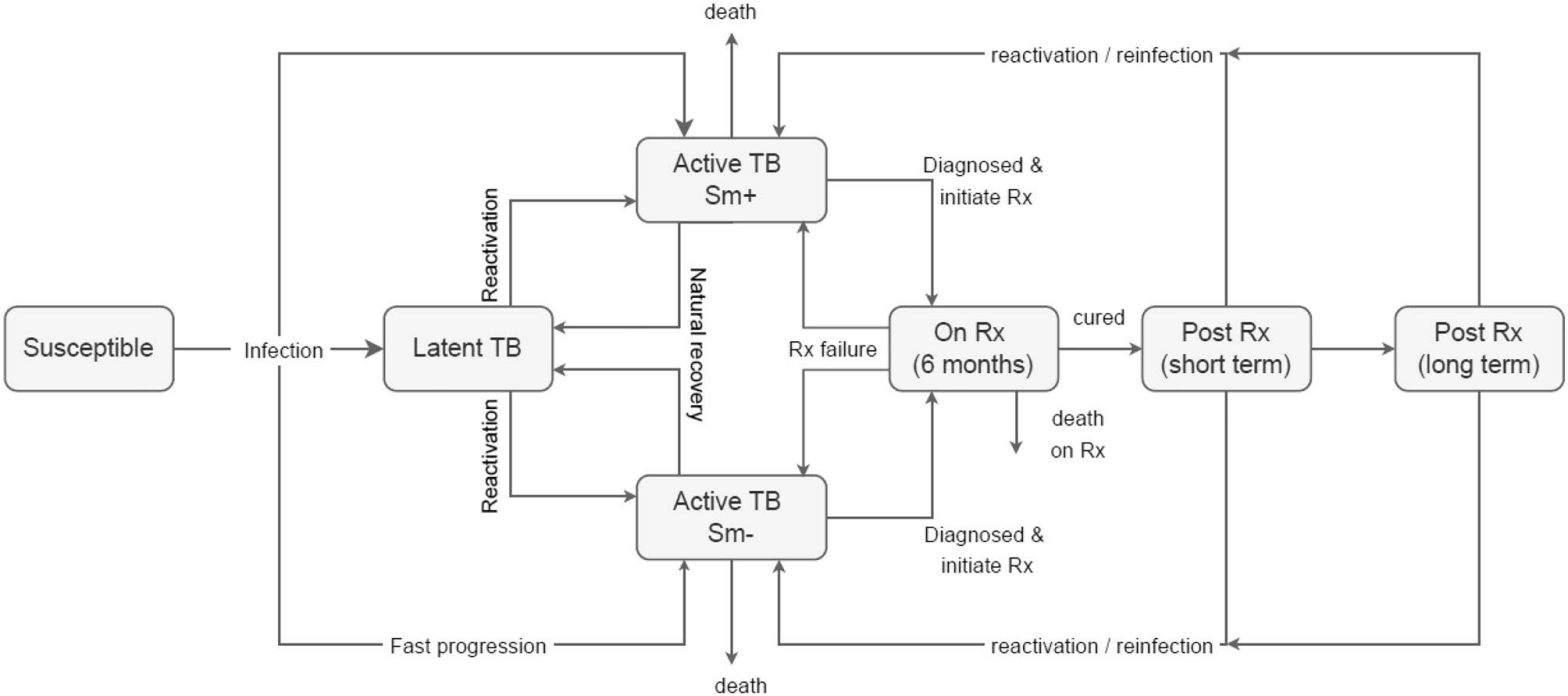
The tuberculosis natural history model structure. TB = tuberculosis. Rx = treatment. Sm+  = smear-positive. Sm− = smear negative. Non-TB mortality transitions are not shown in the figure, but are the same for all states.

We developed an age-sex-stratified deterministic compartmental model of the tuberculosis and HIV epidemics for the South African adult population (aged 15 + years). The core tuberculosis states were modelled following conventions described by previous studies^19^. The risk of infection depends on the mean contact rates, proportions of contacts in each age and sex group^24^, the probability of transmission per contact, and the prevalence of infectious tuberculosis. Transitions between states include tuberculosis infection, progression to tuberculosis disease, natural recovery, diagnosis and treatment initiation (Fig. 1). The outcomes we modelled included cure and treatment failure, and death on treatment, where the rates were estimated from electronic tuberculosis treatment register (ETR) for drug-susceptible tuberculosis (which used different definitions, as shown in Supplementary table 11)^25–27^. Following cure by tuberculosis treatment, two post-treatment states are defined: short-term (within six months after cure) and long-term (six or more months after cure). In both states, individuals are at risk of reinfection, whereas in the short-term post-treatment state, individuals are at a greater risk of recurrent TB due to relapse^28^.

The tuberculosis model was integrated within the Thembisa HIV model^29^. The model is also age-sex-stratified, and the HIV epidemic is simulated dynamically from 1985. HIV-infected sub-populations are further stratified by HIV testing history, CD4 count, and duration since ART initiation. This model also captures changes in the ART guidelines over time and is calibrated to South African HIV data^29^. HIV is assumed to affect the tuberculosis natural history parameters. These HIV effects are modelled as relative risks, depending on CD4 count and receipt of ART.

To capture age and sex differences in tuberculosis incidence, we applied the cumulative multiplicative effect of selected risk factors (alcohol abuse, smoking, undernutrition, and poorly controlled diabetes) to rates of progression to tuberculosis disease. We defined undernutrition as having a body mass index (BMI) < 18.5 kg/m^2^^30^, smoking as those currently smoking tobacco products and accounted for the effects of current smoking and duration of smoking^31^, alcohol abuse as consuming at least 40 g of alcohol on a single day^8^, and diabetes as having HbA1c > 6.5% or Fasting Blood Glucose > 120 mg/dl^30^. These risk factors were selected based on evidence for their effect on developing tuberculosis disease and data reflecting their relatively high prevalence in South Africa (supplementary material). We obtained the age-sex-stratified prevalence of these risk factors from surveys^7,30^. Estimates for the relative effect of these risk factors were obtained from published studies^8–11,31^ and were varied in the calibration process to account for uncertainty around them.

The assumed health-seeking patterns in the model were based on South African studies. We assumed different health facility attendance rates for individuals: 1) with tuberculosis, attending health facilities due to tuberculosis-related symptoms; 2) without tuberculosis, attending due to other health conditions; and 3) without tuberculosis, attending due to tuberculosis-like symptoms. In addition, we assumed that females were more likely to seek care than males^3^, HIV-infected individuals had higher health seeking rates than HIV-uninfected individuals^32^, and smear-positive individuals experienced more tuberculosis symptoms than smear-negative individuals^33^. We also modelled the specificity and sensitivity of the diagnostic algorithm implemented in the model.

Once individuals start the six-month tuberculosis treatment course, the following outcomes were considered: cure, failure, discontinuation, and death. Males were assumed to have higher treatment discontinuation rates than females. Although the base rates of tuberculosis mortality on treatment were initially set the same in males and females, these base rates were adjusted to reflect sex differences in health-seeking patterns. We based treatment outcome assumptions on the electronic tuberculosis treatment register (ETR.net) (supplementary material)^27^.

### Calibration

We used a Bayesian approach to calibrate the model. Prior distributions were set to represent uncertainty in key model parameters (Table 1), and other parameters were fixed at values estimated in earlier model calibrations (supplementary material)^34^. The main data sources used as calibration targets included sex-stratified recorded numbers of tuberculosis deaths from the vital register for 1997–2016; the ETR for sex-stratified numbers of people initiating drug-susceptible tuberculosis treatment (2004–2016), deaths on treatment (2004–2016), and HIV prevalence in treated tuberculosis patients (2008–2016). We also relied on the National Institute for Communicable Diseases for the number of microbiological tests performed (2004–2012)^15^. Lastly, we also used the active tuberculosis prevalence data (2018)^23^.

**Table 1:** Key model parameters.

Parameter description	Mean	Standard deviation	Varied / fixed	Section described in supplementary material
The proportion of incident TB cases in HIV-negative adults that were smear-positive	0.51		Fixed	3
TB transmission probability per contact per day (if an infectious individual was smear-positive)	0.0025	0.0025	Varied	4
The annual rate of reactivation in HIV-negative individuals	0.00148		Fixed	5
The proportion of individuals experiencing fast progression	0.112		Fixed	5
Proportion reduction in TB incidence in previously infected individuals (HIV-negative)	0.79		Fixed	5
Relative rate of immunity to TB per 100-cell increase in CD4	1.1		Fixed	5
Relative rate TB incidence per 100-cell increase in CD4	0.703		Fixed	5
Annual recovery rate in smear-positive TB, HIV-negative individuals	0.075		Fixed	5
Annual recovery rate in smear-negative TB, HIV-negative individuals	0.224		Fixed	5
Relative rate of infectivity: smear-negative compared to smear-positive	0.206		Fixed	5
Annual Smear-negative TB mortality rate (untreated)	0.049		Fixed	5
Annual Smear-positive TB mortality rate (untreated)	0.196		Fixed	5
Relative rate of TB incidence on ART (controlling for CD4)	0.81	0.05	Varied	5
Prevalence of cough > 2 weeks duration in individuals with smear-negative TB	0.198		Fixed	6
Ratio of symptom prevalence in patients with smear-positive compared to smear-negative TB	3.03		Fixed	6
The annual rate of health-seeking in males with smear-negative TB	1.07		Fixed	6
The annual rate of health-seeking in males in the general population	1.0		Fixed	6
The annual rate of health-seeking in males due to TB-like symptoms	0.196		Fixed	6
The proportion of active TB cases seeking treatment who are treated empirically before any microbiological test is done	0.271		Fixed	6
The proportion of smear-negative TB cases which ae treated empirically if they initially screened negative on a smear test	0.423		Fixed	6
Relative rate of empirical treatment if not seeking treatment because of TB symptoms	0.031		Fixed	6
Relative rate empirical treatment if symptoms are not due to TB	0.0014		Fixed	6
Reduction in empiric treatment after a negative screen due to Xpert MTB/RIF	0.50		Fixed	6
Relative rate of health-seeking in women, compared to men	1.55	0.17	Varied	6
Relative rate of health-seeking in HIV-positive compared to HIV-negative individuals	4.27		Fixed	6
Relative rate of screening in TB patients seeking treatment for TB symptoms, compared to those seeking treatment for other conditions: initial^§^	11.10		Fixed	6
Relative rate of screening in TB patients seeking treatment for TB symptoms, compared to those seeking treatment for other conditions: final^§^	3.84		Fixed	6
Probability of cure if a patient dropped out before completing TB treatment	0.65		Fixed	7
Increase in TB mortality rate per 10-year increase in age	1.4	0.1	Varied	7
The annual mortality rate in HIV-negative individuals receiving TB treatment⁋	0.192		Fixed	7
The relative rate of TB mortality per 50 cell increases in CD4 count if HIV +	0.87	0.05	Varied	7
Relative rate of TB mortality if on ART	0.55	0.08	Varied	7
Increase in TB risk if previously experienced TB	3.03		Fixed	8
Annual rate of relapse in short term post-treatment state	0.1		Fixed	8
Increase in TB incidence due to alcohol abuse	1.94†	0.65	Varied	10
Increase in TB incidence due to diabetes (HbA1c > 6.5%)	2.59†	0.83	Varied	10
Increase in TB risk if currently smoking	0.47†	0.39	Varied	10
Increase in TB risk per 10-year increase in the duration of smoking	0.38†	0.12	Varied	10
Increase in TB risk due to low BMI	0.8†	0.25	Varied	10

ART = antiretroviral therapy; BMI = body mass index; HbA1c = Glycated haemoglobin; TB = tuberculosis. Additional details and references on the parameter values are provided in the supplementary material. ⁋Applies to when most people get treated in the very advanced stages of disease (i.e., when screening rates are very low, close to zero). †A value of 2.59, for example, is equivalent to an RR of 3.59 when comparing individuals with the exposure to individuals in the baseline category. Similarly, a value of 0.39 is equivalent to an RR of 1.39 when comparing individuals with the exposure to individuals in the baseline category. ^§^This is a time-varying parameter. The initial rate applies up to 2005 (initial), then we estimate a rate that applies from 2012 with linear interpolation over the intervening years (ultimate).

For the calibration process, likelihood functions were defined to represent the goodness of fit to these calibration targets, allowing for possible under- or over-reporting in the vital register and the ETR data. We simulated posterior distributions numerically using Incremental Mixture Importance Sampling. Importance sampling was used to draw a sample of parameter combinations from regions of the parameter space that yielded the highest likelihood values to generate posterior estimates^35^. The means for the model estimates were calculated from 1000 posterior samples, and 95% confidence intervals were calculated by taking the 2.5th and 97.5th percentiles of the posterior sample (supplementary material).

### Model experiments and outcomes

We estimated M:F ratios for tuberculosis incidence and mortality using the model-estimated rates of new tuberculosis cases and deaths under the baseline scenario (A) which represents the actual tuberculosis and HIV epidemic up to 2019, incorporating sex differences.

To assess the effect of a specific factor on tuberculosis incidence and mortality, and the M:F ratios, we ran individual counterfactual scenarios (B1-11) where each factor was excluded or set equal in males and females in the model, and then compared the model outputs to the outputs obtained in the baseline scenario (A) (Table 2). We calculated sex-stratified percentage increases in tuberculosis incidence and mortality due to HIV; percentage decreases in tuberculosis incidence and mortality due to ART; and tuberculosis incidence PAFs due to smoking, alcohol abuse, undernutrition, diabetes, and HIV. Lastly, we calculated percentage changes in M:F ratios for tuberculosis incidence and mortality under the baseline compared to all counterfactual scenarios.

**Table 2:** Model scenarios to quantify the extent to which various factors contribute to sex differences in tuberculosis.

Model scenarios	Description
Baseline scenario (A)	Baseline scenario which represents the actual tuberculosis and HIV epidemic up to 2019, incorporating sex differences
Counterfactual scenarios (B)	
1. No HIV epidemic	HIV transmission probabilities were set to zero
2. No ART	Annual rates of ART initiation are set to zero
3. Equal ART uptake	Annual HIV testing and ART initiation rates in males and females assumed to be the same
4. No smoking	Prevalence of smoking is zero
5. No alcohol abuse	Prevalence of alcohol assumption is zero
6. No undernutrition	Prevalence of undernutrition is zero
7. No diabetes	Prevalence of diabetes is zero
8. Equal health seeking	TB health seeking rates for females are set the same as for males
9. Equal social mixing	Contact rates and social mixing parameters are set as the average of the baseline male and female parameters
10. Equal treatment discontinuation	Treatment discontinuation in males set the same as for females
11. All effects equal	Assume no HIV epidemic and all the other parameters for the factors above are set the same for males and females

ART = antiretroviral therapy; TB = tuberculosis.

### Ethics

This modelling analysis relied on aggregated data drawn from publicly available data sources which are presented in the supplementary material, and human ethics review was not required.

## Results

**Figure 2:**
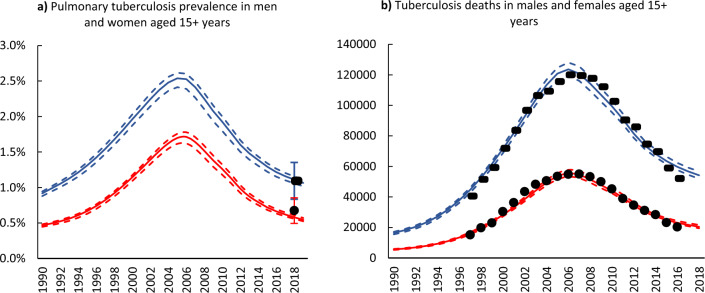
Sex-specific tuberculosis prevalence and mortality in adults, 1990–2019. (**a**) Blue and red solid lines represent model estimates for tuberculosis prevalence in males and females, respectively. Black dots represent the 2018 TB prevalence survey with 95% confidence intervals. (**b**) Blue and red solid lines represent model estimates for tuberculosis mortality in males and females, respectively. Black dots represent recorded mortality, adjusted for the cause of death misclassification and missing fields. All dashed lines represent 95% confidence intervals.

Overall, the model estimates for tuberculosis prevalence and mortality were consistent with the sex-stratified observed data, with a higher burden in males than females (Fig. 2a,b). Tuberculosis prevalence and deaths rose rapidly during the early 1990s, peaked in the mid-2000s to late-2000s, and subsequently declined until 2019. The 2019 estimated tuberculosis prevalence in males was 1.06% (95% CI 1.0–1.12%) and 0.58% (95% CI 0.56–0.62%) in females. Tuberculosis deaths in 2019 were 32 000 (95% CI 29 000–35 000) in males and 21 000 (95% CI 19 000–22 000) in females.

**Figure 3:**
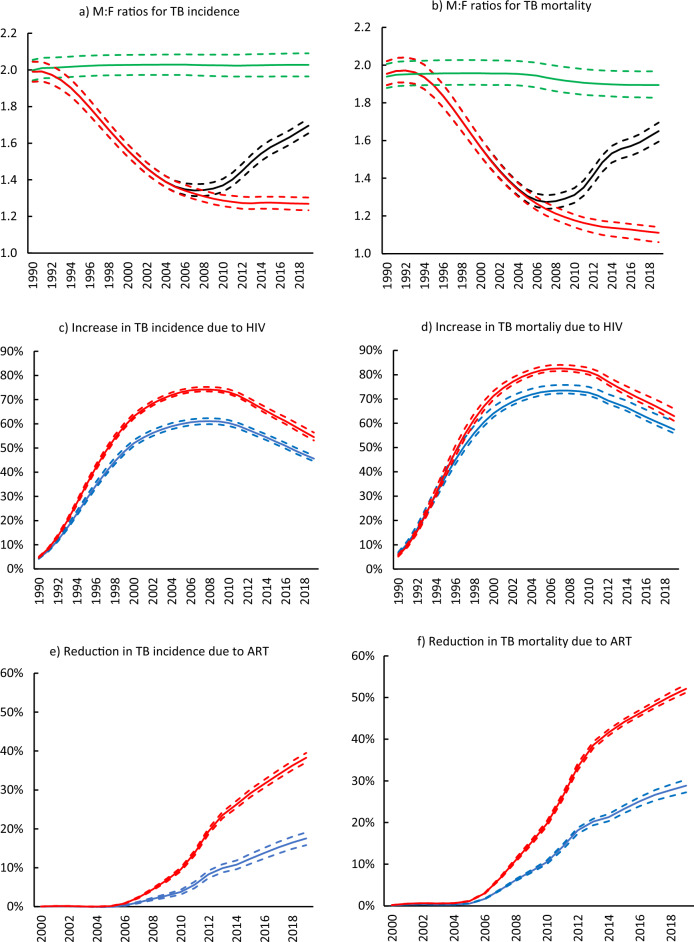
The effect of HIV and ART on male-to-female (M:F) ratios for tuberculosis incidence and mortality, 1990–2019. First row: M:F ratios for tuberculosis incidence (**a**) and mortality (**b**) (1990–2019). The solid black lines represent the baseline scenario where the effects of HIV and the rollout of ART from the year 2000 were present in the model. The solid green lines represent the counterfactual scenario where HIV was absent from the model. The solid red lines represent the counterfactual scenario where HIV was present, but ART was not introduced. Second row: Percentage increase in tuberculosis incidence (**c**) and mortality (**d**) due to HIV. The solid red lines represent female tuberculosis incidence and mortality, and the solid blue lines represent males. Third bottom row: Percentage reduction in tuberculosis incidence (**e**) and mortality (**f**) due to ART. All dashed lines represent 95% confidence intervals. ART = antiretroviral therapy; TB = tuberculosis.

Over the 1990–2019 period, the M:F ratios for tuberculosis mortality and incidence were consistently greater than 1.0. The M:F ratios for tuberculosis incidence and deaths were the highest in the early 1990s (Fig. 3a,b, black); if HIV had not been present in South Africa, the M:F ratios would have remained relatively stable, at around 2.0 (Fig. 3a,b, green). As the HIV epidemic rapidly grew in the South African population, tuberculosis mortality and incidence for both sexes increased (1996–2002). However, females had a more substantial increase in tuberculosis incidence and mortality due to HIV than males (Fig. 3c,d). Consequently, the M:F ratios for tuberculosis incidence and mortality declined and reached their lowest points in the mid-2000s to late-2000s (Fig. 3a,b, black).

If ART had not been rolled out in South Africa, the M:F ratio for tuberculosis incidence and mortality would have continued to decline (Fig. 3a,b, red dotted). However, with the expansion of ART from the mid-2000s, the M:F ratios increased, reaching 1.70 (95% CI 1.65–1.73) for incidence and 1.65 (95% CI 1.59–1.70) for mortality in 2019.

Over time, HIV led to a greater relative increase in tuberculosis incidence and mortality in females than males. In 2019, HIV contributed to a 54.0% (95% CI 53.1–56.4%) and 62.9% (95% CI 60.9–66.0%) increase in female tuberculosis incidence and mortality, respectively (Fig. 3c,d, red). Among males, HIV led to 45.6% (95% CI 44.5–46.1%) increase in tuberculosis incidence and 57.4% (95% CI 55.8–60.7%) increase in tuberculosis mortality (Fig. 3c,d; blue). However, females benefited more from ART than males. In 2019, ART resulted in 38.3% (95% CI 37.1–39.5%) and 52.1% (95% CI 51.2–53.1%) reductions in tuberculosis incidence and mortality among females, respectively (Fig. 3e,f, red). For males, ART led to 17.5% (95% CI 15.8–19.1%) and 28.8% (95% CI 27.3–30.3%) reductions in tuberculosis incidence and mortality, respectively, in 2019 (Fig. 3e,f, blue).

**Figure 4:**
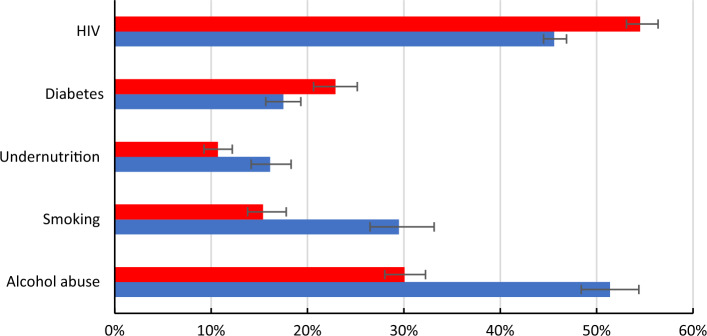
Population attributable fractions for tuberculosis incidence in 2019 due to alcohol abuse, smoking, undernutrition, diabetes, and HIV. The red bars represent females, and the blue bars represent males.

The PAFs of tuberculosis incidence due to alcohol abuse, smoking, and undernutrition were higher in males than females, estimated at 51.4% (95% CI 48.4–54.4%), 29.5% (95% CI 26.5–33.1%) and 16.1% (95% CI 14.1–18.3%) respectively among males in 2019 (Fig. 4); among females, the estimated PAFs were 30.1% (95% CI 28.0–32.2%), 15.4% (95% CI 13.8–17.8%) and 10.7% (95% CI 9.3–12.2%) respectively. On the other hand, the PAFs of tuberculosis incidence due to diabetes and HIV were higher in females (22.9% (95% CI 20.6–25.2%) and 54.5% (95% CI 53.1–56.4%) respectively) than in males (diabetes: 17.5% (95% CI 15.7–19.3%) and HIV: 45.6% (95% CI 44.5–46.9%).

In the counterfactual scenario where HIV was assumed absent, and all other factors' effects were set equal for males and females, the tuberculosis incidence M:F ratio reduced from 1.7 to 1.01, relative to the baseline scenario (40.7% reduction); for mortality, the M:F ratio reduced from 1.65 to 0.93, (43.6% reduction) (Table 3). In the counterfactual scenario where we assumed equal annual rates of HIV testing and ART initiation in males and females, the tuberculosis incidence and mortality M:F ratios reduced by 3.4% and 8.2%, respectively. When we assumed no ART, the tuberculosis incidence and mortality M:F ratios reduced by 25.1% and 32.7%, respectively. In the individual counterfactual scenarios where alcohol abuse, smoking and undernutrition were excluded, the tuberculosis incidence M:F ratios reduced by 30.5%, 16.8% and 6.1%, respectively. On the other hand, excluding HIV and diabetes led to 19.6% and 7.0% increases in the M:F ratios, respectively.

**Table 3:** Male-to-female ratios for TB incidence and mortality in the baseline and counterfactual scenarios, 2019.

	TB incidence M:F ratios (95% CI)	% change in M:F ratio, counterfactual vs baseline (95% CI)	TB mortality M:F ratios (95% CI)	% change in M:F ratio, counterfactual vs baseline (95% CI)
Baseline scenario	1.70 (1.65–1.73)		1.65 (1.59–1.66)	
Counterfactual scenarios				
1. No HIV epidemic	2.03 (1.20–2.10)	$\uparrow$ 19.6 (17.5–21.8)	1.89 (1.83—1.97)	$\uparrow$ 14.8 (12.3–17. 5)
2. No ART	1.27 (1.23–1.30)	$\downarrow$ 25.1 (24.4–26.1)	1.11 (1.06—1.14)	$\downarrow$ 32.7 (31.5–34.1)
3. Equal ART uptake	1.64 (1.46–1.68)	$\downarrow$ 3.4 (2.9 –3.9)	1.52 (1.46—1.56)	$\downarrow$ 8.2 (7.6–8.7)
4. No smoking	1.41 (1.37–1.45)	$\downarrow$ 16.8 (14.7–18.6)	1.36 (1.31—1.40)	$\downarrow$ 17.9 (15.9–20.0)
5. No alcohol abuse	1.18 (1.14–1.22)	$\downarrow$ 30.5 (28.2–32.7)	1.08 (1.04—1.11)	$\downarrow$ 34.8 (32.7–36.8)
6. No undernutrition	1.59 (1.55–1.63)	$\downarrow$ 6.1 (5.3–7.0)	1.56 (1.52—1.61)	$\downarrow$ 5.4 (4.6–6.2)
7. No diabetes	1.81 (1.77–1.86)	$\uparrow$ 7.0 (6.2–7.8)	1.80 (1.74—1.85)	$\uparrow$ 8.9 (7.8–9.9)
8. Equal health seeking	1.75 (1.70–1.78)	$\uparrow$ 2.9 (2.5–3.4)	1.52 (1.48—1.56)	$\downarrow$ 7.7 (6.8–8.9)
9. Equal social mixing	1.75 (1.70 –1.79)	$\uparrow$ 3.3 (3.02–3.6)	1.70 (1.64—1.75)	$\uparrow$ 2.9 (2.7–3.2)
10. Equal treatment discontinuation	1.70 (1.65–1.73)	$\uparrow$ 0.015 (0.004–0.026)	1.66 (1.60—1.70)	$\uparrow$ 0.45 (0.42–0.48)
11. All effects equal^(a)^	1.01 (1.00–1.01)	$\downarrow$ 40.7 (39.1–42.0)	0.93 (0.92—0.94)	$\downarrow$ 43.6 (41.7–45.2)

ART = antiretroviral therapy. CI = confidence interval. M:F ratio = male-to-female ratio. $\downarrow$=decrease. $\uparrow$=increase. Under the baseline scenario, all factors were included in the model. Individual counterfactual scenarios were simulated with the exclusion of specific factors (HIV, alcohol, smoking, undernutrition, and diabetes). (3): equal ART scenario = annual rates of HIV testing and ART initiation in males and females are set the same; (8): health-seeking rates for females set the same as for males; (9): contact rates and social mixing parameters set to be the average of the baseline male and female contact rates; (10): rates of treatment discontinuation in males set to be the same as for females. Under the counterfactual scenarios (11): ‘All effects equal’ = HIV epidemic assumed absent and all the other parameters for the factors above were set the same for males and females, with no effects of alcohol, smoking, undernutrition, and diabetes on TB.

The counterfactual scenario for health-seeking patterns (equal male and female rates) led to a 7.7% decrease in the M:F ratio for TB mortality. When social contact rates were the same in males and females, the M:F ratios for tuberculosis incidence and mortality slightly increased by 3%. Lastly, in the treatment discontinuation counterfactual scenario (male discontinuation rate set at the same value as female rate), there were negligible changes in the M:F ratios for tuberculosis incidence and mortality.

## Discussion

Our model suggests that despite variations in the M:F ratios for tuberculosis incidence and mortality over the 1990–2019 period, overall tuberculosis mortality and incidence were consistently higher in males than females. The higher tuberculosis incidence in males may partly be explained by alcohol abuse and smoking, which are highly prevalent in males and increase the risk of developing tuberculosis through weakening cell-mediated immunity^11,12^. Low ART uptake among men compared to women also explains the excess burden of tuberculosis in males. ART substantially reduced the contribution of HIV to tuberculosis incidence in both sexes; however, higher levels of HIV testing and ART initiation among females compared to males^36^ led to females experiencing greater relative reductions in tuberculosis due to ART than males. We also showed that health-seeking delays explain the higher mortality among males, while sex differences in social mixing patterns and treatment discontinuation had minor effects on sex disparities in tuberculosis.

HIV had a greater effect on tuberculosis incidence and mortality among females than males due to the higher HIV prevalence in females^13^. Consequently, the M:F ratios for tuberculosis incidence and mortality declined during the mid-1990s to early 2000s as HIV was rapidly increasing. However, the expansion of the ART program substantially reduced tuberculosis incidence and mortality, and the higher levels of ART coverage in women compared to men^36^ have meant that male tuberculosis rates have not declined to the same extent as those in women. These findings are consistent with other studies demonstrating that although HIV prevalence was higher among females, males still had a greater burden of tuberculosis than females^15,17^. Hermans and colleagues showed that HIV led to substantial relative increases in tuberculosis notification rates among females than males between 1993 and 2013; and the scale-up of ART led to substantial declines in females' relative tuberculosis notification rates compared to males^17^. Altogether, the modelled HIV and ART effects on the sex distribution of tuberculosis support the hypothesis that if HIV removed the protection females have against tuberculosis disease^5^, ART restored this protection ^17^.

In contrast with our tuberculosis incidence estimates for 2019, the GBD study estimated higher tuberculosis incidence in females than males for South Africa^2^. This disparity was attributed to the higher burden of HIV in females than in males ^2,13^. Differences in methodological approaches and data sources may explain these discrepancies between our estimates and those by the GBD. For instance, the GBD used meta-regression models and relied on mortality data to estimate tuberculosis incidence from mortality-incidence ratios^2^. In contrast, we used a dynamic transmission model which accounts for the tuberculosis natural history, impact of HIV and interventions.

Nonetheless, our tuberculosis mortality estimates were consistent with the GBD estimates, with higher mortality in males than females. The GBD suggested that the excess tuberculosis mortality in males was mainly due to alcohol abuse and smoking among HIV-negative individuals^2^. We also found alcohol and smoking to be important contributors to the overall tuberculosis incidence and sex differences. Tuberculosis incidence PAFs due to smoking and alcohol abuse were higher in males than females, and we demonstrated that if smoking or alcohol abuse were removed individually, M:F ratios for tuberculosis incidence would reduce by approximately 17% or 30%, respectively. This reflects the increased exposure males have to these risk factors, which are also likely to increase the progression to tuberculosis disease^11,12^. On the other hand, because HIV and diabetes are relatively more prevalent in females than males, females had higher PAFs for HIV and diabetes.

In the counterfactual scenario where males' health-seeking rates were increased and set equal to female health-seeking rates, the overall tuberculosis incidence and mortality declined slightly. This counterfactual scenario was associated with a 7% reduction in the M:F ratio for tuberculosis mortality, suggesting that delays to diagnosis and treatment in males may lead to tuberculosis disease severity and death^37^. Supporting these findings, other studies suggested that compared to females, males are older and sicker when they seek health care^3^; they are more likely to be lost to follow-up and experience poor outcomes, including treatment failure and death^19,37,38^. The health-seeking delays in men may be explained by socioeconomic reasons such as the higher rates of employment in men and associated loss of income due to time lost while seeking tuberculosis health care^38^.

The assumed social mixing counterfactual scenario modestly influenced the tuberculosis incidence and mortality M:F ratios. In this scenario, social mixing proportions and contact rates were the same in men and women. The model estimated slight increases in tuberculosis incidence and mortality in females and declines in males, and the M:F ratios increased moderately. This is due to the higher contact rates in women that we assumed in the baseline scenario. However, in other studies where the social mixing patterns were assumed to be highly sex-assortative, sex disparities in tuberculosis increased^20,22^. This was mainly due to men having higher rates of social contact with other men who carry a higher tuberculosis prevalence and therefore further increasing the risk of transmission and the burden of tuberculosis among men^20,22^.

Our model suggests that removing an individual risk factor or equalising males' and females' health-seeking patterns, social contacts, or treatment outcomes at the current levels is insufficient to eliminate sex disparities. However, assuming the HIV epidemic was absent and all the other factors in males and females were the same, the tuberculosis incidence M:F ratios were reduced to 1.01. Some of the remaining differences may be due to other factors such as biological differences that we did not model. For mortality, the M:F ratio reduced to 0.93 (i.e., higher mortality in females). This is possibly because females’ ‘background’ mortality (deaths not related to HIV and tuberculosis, e.g., due to violence) is much lower than in males^27,39^; and therefore, more females survive to older ages than males. In our model we assumed tuberculosis mortality rates increase with age (Table 1), people in older age groups (55+ years) contribute disproportionately to tuberculosis mortality, and the majority are likely females. The lower female background mortality rates may also explain why in females compared to males, tuberculosis mortality may appear higher relative to background mortality^27^, although their absolute tuberculosis mortality risk is low.

Our analysis is strengthened by using a tuberculosis and HIV transmission model calibrated to several South African data sources. This dynamic model allowed us to quantify how HIV and ART affected the sex distribution of tuberculosis incidence and mortality over the 1990–2019 period. However, our study has several limitations. First, we did not include all the factors that may drive sex differences in tuberculosis, such as differences in biological susceptibility to tuberculosis disease^5^, occupational exposures such as mining, or incarceration^40,41^. Second, we did not model alcohol, smoking, undernutrition and diabetes dynamically; their effects depended on their prevalence in the population, with most of the prevalence estimates calculated from 2016 data^7,30^. The prevalence of these risk factors may have changed over time. Hence, the estimated PAFs (for 2019) may not accurately represent the historical effect of these risk factors on tuberculosis incidence. For instance, the prevalence of smoking has been on a declining trend^42^, and diabetes has risen over time^43^. Another limitation is that it is not clear whether these risk factors affect tuberculosis transmission, the incidence of tuberculosis disease or mortality. However, for simplicity, we have modelled only the effect of these risk factors on tuberculosis incidence. Lastly, we acknowledge the limitation that although some evidence suggests there are sex differences in latent tuberculosis infection (LTBI)^44^ we did not model sex differences in LTBI, this is partly due to the lack of South African based data on the prevalence of LTBI by sex.

In summary, men have consistently had higher tuberculosis incidence and mortality than women. The excess tuberculosis incidence and mortality in men highlights the need to make health services more accessible to men and address the structural barriers to their retention in tuberculosis and HIV care. Additionally, there is a need for effective interventions that reduce excessive alcohol consumption and tobacco smoking. Lastly, TB incidence can be reduced through better prevention and treatment of diabetes, especially in women^45^.

## Supplementary Information

Supplementary Information.

## Data Availability

All data generated or analysed during this study are included in this published article (and its Supplementary Information files).
